# Process evaluations of task sharing interventions for perinatal depression in low and middle income countries (LMIC): a systematic review and qualitative meta-synthesis

**DOI:** 10.1186/s12913-018-3030-0

**Published:** 2018-03-23

**Authors:** Memory Munodawafa, Sumaya Mall, Crick Lund, Marguerite Schneider

**Affiliations:** 1 Alan J Flisher Centre for Public Mental Health, Department of Psychiatry and Mental Health, University of Cape Town, 46 Sawkins Road, Rondebosch, Cape Town, South Africa; 2 Department of Psychiatry and Mental Health, Faculty of Health Sciences, University of Cape Town, Cape Town, South Africa; 3 Division of Epidemiology and Biostatistics, School of Public Health, Faculty of Health Sciences, University of Witwatersrand, Johannesburg, South Africa; 4 King’s College London, Centre for Global Mental Health, Institute of Psychiatry, Psychology and Neuroscience, London, UK

**Keywords:** MRC process evaluation guidelines, Perinatal depression, Task shared intervention, Lay health worker, Low and middle income country

## Abstract

**Background:**

Perinatal depression is common in low and middle income countries (LAMICs). Task sharing interventions have been implemented to treat perinatal depression in these settings, as a way of dealing with staff shortages. Task sharing allows lay health workers to provide services for less complex cases while being trained and supervised by specialists. Randomized controlled trials suggest that these interventions can be effective but there is limited qualitative information exploring barriers and facilitators to their implementation. This systematic review aims to systematically review current qualitative evidence of process evaluations of task sharing interventions for perinatal depression in LAMICs in relation to the United Kingdom (UK) Medical Research Council (MRC) framework for conducting process evaluations.

**Methods:**

We searched Medline/ PubMed, PsycINFO, Scopus, Cochrane Library and Web of science for studies from LAMICS using search terms under the broad categories of: (a) “maternal depression’” (b) “intervention” (c) “lay counsellor” OR “community health worker” OR “non-specialist” and (d) “LAMICs”. Abstracts were independently reviewed for inclusion by two authors. Full text articles were screened and data for included articles were extracted using a standard data extraction sheet. Qualitative synthesis of qualitative evidence was conducted.

**Results:**

8420 articles were identified from initial searches. Of these, 26 full text articles were screened for eligibility with only three studies meeting the inclusion criteria. Main findings revealed that participants identified the following crucial factors: contextual factors included physical location, accessibility and cultural norms. Implementation factors included acceptability of the intervention and characteristics of the personnel. Mechanisms included counsellor factors such as motivating and facilitating trust; intervention factors such as use of stories and visual aids, and understandability of the content; and participant factors such as shared experience, meeting learning needs, and meeting expectations.

**Conclusions:**

While task sharing has been suggested as an effective way of filling the treatment gap for perinatal depression, there is a paucity of qualitative research exploring barriers and facilitators to implementing these interventions. Qualitative process evaluations are crucial for the development of culturally relevant interventions.

**Electronic supplementary material:**

The online version of this article (10.1186/s12913-018-3030-0) contains supplementary material, which is available to authorized users.

## Background

Perinatal depression is a significant public health issue in both high income (HIC) and low and middle income countries (LAMICs) [1, 2]. Perinatal depression which refers to the experience of depression during pregnancy and up to one year post-partum can be associated with adverse consequences for both mother and baby [3]. In LAMICs, where resources are few, and access to mental health professionals is limited, [4–7] prevalence of perinatal depression is estimated to be 15.9% [8]. The experience of perinatal depression in LAMICS is exacerbated by poverty, unemployment, HIV/AIDS, and intimate partner violence.

LAMICS have a “treatment gap” where up to 75% of people who need mental health treatment do not always receive optimal care [9, 10]. Research suggests that task sharing is a successful means of addressing this “treatment gap” for perinatal depression in resource poor settings [11, 12]. Task sharing is an approach to mental health service provision whereby non-specialist health workers provide care for less complex cases under the training and supervision of a specialist. This shares the burden of care [4] while providing locally relevant interventions to people from the same community and cultural background who speak the same language [13, 14]. Although the line between efficacy (whether the treatment works under ideal circumstances) and effectiveness (whether the treatment works in real world situations) [15] in relation to task sharing for mental health in LAMICS has been somewhat blurred, many trials have proceeded to effectiveness evaluations without necessarily demonstrating efficacy. Several studies have suggested that task sharing has benefits and results from a Cochrane review indicate that non-specialist workers can be trained to deliver psychological interventions with training and supervision in order to improve the symptoms of perinatal depression in mothers [16]. Task sharing has also been shown to be effective for the treatment of perinatal depression in Pakistan and for depression in men and women in Uganda [17, 18]. There is a need for both process evaluations and in-depth qualitative analyses of task shared interventions to develop a better understanding of factors contributing to their sustainability. Providing qualitative evidence on interventions is crucial for gaining insight into the participants’ and service providers’ views on the development of acceptable interventions [19].

The new United Kingdom (UK) Medical Research Council (MRC) framework for process evaluations provides guidelines for conducting process evaluations in order to assess the quality of implementation and fidelity to the intervention [20]. The framework further recommends examining the relationship between three main factors: implementation, context and mechanisms [20]. *Implementation* includes examining the resources provided for the intervention and their appropriateness, such as counsellor training, supervision, manuals, dose and reach (the total number of sessions and participants reached) [20]. The *context* includes examining the external environmental or community (such as rural or urban setting and common cultural or religious practices), and service structure factors such as acceptance by local Primary Health Centre [5]. *Mechanisms* refer to participant responses to the intervention and the aspects of the intervention that lead to change in the participant’s behaviour including counsellor motivation to conduct the intervention and participants’ motivation to attend sessions [20]. In this study we understand mechanisms to include both the mechanisms of the intervention and the mechanisms of implementation (which are important to consider in the context of task sharing). The context, implementation and mechanisms can be used to examine factors that affect the intervention [20]. There have been previous qualitative studies on task sharing for mental health care in LAMICS without a clear process evaluation which highlight important factors that affect the intervention’s acceptability and feasibility. These include: service providers’ level of confidence, distress experienced by participants, fidelity to the intervention, acceptability of the intervention, costs and policy alignment and adequate incentives [21]. Several barriers to task shared interventions have been noted including poor adherence, low acceptability of talk therapy, stigma of mental health interventions and burnout due to increased workload for service providers [13]. Synthesising themes across these various studies is useful to evaluate the appropriateness, acceptability and effectiveness of interventions [22, 23]. Qualitative studies can provide nuanced detailed understandings regarding the process of delivering interventions which are not accessible through quantitative data. Within the context of task sharing, qualitative studies can complement quantitative studies because most studies do not report qualitative data from trials, and this is an important area to highlight for future research [24].

To our knowledge no systematic review has been conducted to synthesise qualitative evidence on process evaluations of task shared intervention for perinatal depression in LAMICs. This review seeks to answer two main questions: (i) to what extent are qualitative process evaluations conducted on task shared interventions for perinatal depression in LAMICs; and (ii) what is the best way to synthesize emergent themes from the process evaluations with the MRC framework for conducting process evaluations [20]?

## Methods

The full protocol is registered on the PROSPERO database URL (http://www.crd.york.ac.uk/PROSPERO/display_record.asp?ID=CRD42015025190).

### Search strategy

Five electronic databases were searched between September and December 2015 - Medline/ PubMed, PsycINFO, Scopus, Cochrane Library and Web of science. The search terms included four concepts (a) “maternal depression’” (b) “intervention” (c) “lay counsellor” which were expanded by using “community health worker” OR “non-specialist” and (d) “LAMICs” as determined by the World Bank Country classification. These phrases were adapted for use in each database.The terms “task sharing” and “process evaluation” were excluded from searches since they restricted the number of abstracts identified. In PubMed the following search terms were used, and adapted for use in other databases:

(((((perinatal) OR prenatal) OR antenatal) OR postnatal) OR postpartum) OR post-partum AND depression AND (((((community health workers) OR community health aides) OR village health workers) OR health personnel) OR fieldworkers AND counselling OR psycho-social intervention*AND developing countries.

A full description of the search strategy is included as Additional file 1. The inclusion and exclusion criteria are presented in Table 1.

**Table 1:** Inclusion and Exclusion Criteria

	Inclusion Criteria	Exclusion Criteria
Publication Type	• Qualitative evidence of process evaluations of psycho-social treatment interventions for antenatal or postnatal depression	• Quantitative studies which do not have a qualitative component
Study Design	• Studies which evaluate effectiveness of both pharmacological and psycho-social intervention	• Studies that only evaluate pharmacological interventions
Condition of Interest	• Antenatal OR Post-natal OR Perinatal depression	• Studies of other conditions which are not perinatal depression
Type of intervention	Psycho-social counselling or psychoeducation	• Studies that do not include counselling or psychoeducation
Time point	• Post-intervention evaluation	• Pre-intervention evaluation
Study Population	• Group and individual intervention by non-specialists	• Studies where intervention is conducted by mental health specialists
Intervention Location	• Studies in LAMICS	• Studies in HICs
Language	Studies in English	Studies not in English

Abstracts identified were imported into Endnote and duplicates were removed. MM (primary reviewer) and SM (secondary reviewer) independently reviewed the abstracts for each paper using the eligibility criteria described in Table 1. Upon initial screening, the majority of the articles were excluded for the following reasons; not an intervention study, a review paper not intervention and treatment other than counselling. Once full-text articles had been retrieved, MM and SM independently reviewed the studies again and the following criteria were used to further exclude papers such as: studies that do not employ a qualitative methodology, were not process evaluations nor task shared interventions and did not target perinatal depression. MM and SM had several face to face discussions to reach consensus on studies. In cases where studies provided limited information on the intervention, the authors were contacted to provide further information.

### Quality appraisal

The review used the 2009 Preferred Reporting Items for Systematic reviews and Meta-Analyses (PRISMA) statement which ensures that the study reported fits the reporting standards of systematic reviews, assesses the quality, structure and whether there is a clear explanation of the objectives, methods and results [25]. The PRISMA Statement is included as Additional file 2. Data were extracted using a standard data extraction table which included the following: date of publication, setting of the study (hospital/ clinic/community), study design, number of participants, age range, measures used, validity of measures, quality assessment and main process evaluation findings. This table is provided in Additional file 3. The quality of the included studies was assessed by both reviewers independently using the Critical Appraisal Skills Programme (CASP) checklist which examines risk of bias, and whether the study design, recruitment strategy, data collection and analysis were appropriate for the study [26]. The CASP checklist is provided in Additional file 4.

### Data analysis and meta-synthesis

Data analysis was conducted using thematic analysis. The reviewers followed the 3 steps set out by Thomas and Harden [27]: (i) free coding of data (ii) organising coded data into descriptive themes and lastly, (iii) generating analytical themes. The reviewers read the full text articles and conducted free coding of data by reading each line of text and organising the free codes into hierarchical groups of descriptive themes based on their similarities or differences [27]. Meta-synthesis involved interpreting, integrating and inferring the process evaluation elements from all the included studies identified and generating hypotheses based on these findings after discussion and consensus among the reviewers [23]. Emerging themes were integrated into the MRC Framework of context, implementation and mechanisms and further classified into sub-themes where applicable.

## Results

**Fig. 1:**
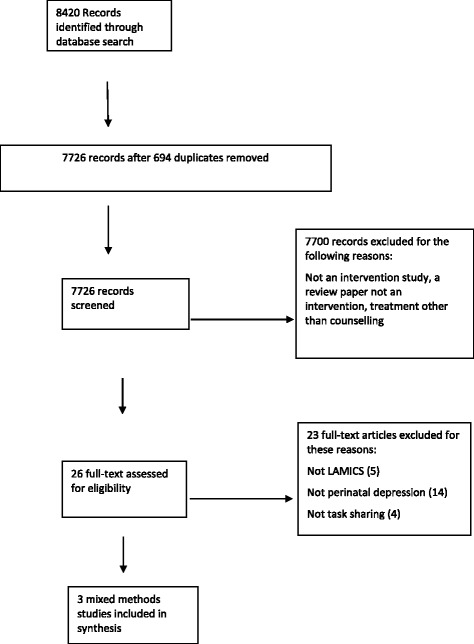
Flow diagram of study selection

The database search identified 8420 articles which were screened per the process outlined in Fig. 1 and 7703 articles were excluded for the reasons set out above.

### Description of the studies

We screened 26 full text articles and three studies were selected for final inclusion in this review. All studies were written in English. The studies included were the Thinking healthy Programme (THP) from Pakistan [28], The Ekjut trial of Participatory Learning Action Groups (PLAG) in India [29] and the Interpersonal Therapy (IPT) trial in China [30]. The THP and Ekjut studies were designed as cluster Randomised Controlled Trials (RCT) [28, 29] and the IPT study was an individual level RCT [30]. Depression was measured by three different scales, the IPT study used the Edinburgh Postnatal Depression Scale (EPDS) [31] with a cut off score of 13 and above, the THP recipients were diagnosed by a trained psychiatrist using the Schedules for the Assessment of Neuropsychiatry (SCAN) [32]. The Ekjut study used the Kessler 10 (K10) [33] with cut offs between 16 and 50 to assess for depression in the second and third year of the study due to difficulty selecting a contextually appropriate scale and did not use depression as part of their initial screening criteria. Findings reported in this current paper are for the intervention groups of the RCTs only.

All three RCTs indicated that the intervention was effective as measured by various outcome measures. The Ekjut study was focused on reducing neonatal deaths and noted a 45% reduction in neonatal mortality in the last two years of the intervention along with a 57% reduction in moderate depression in their third year [34]. The THP reported a reduction in maternal depression at six months postnatal with (23%), 97 out of 418 mothers compared to (53%), 211 out of 400 mothers meeting the criteria for major depression [17]. The IPT focused on preventing postpartum depression (PPD) and reported improved psychological wellbeing, improved interpersonal relationships and fewer symptoms of depression in the intervention compared to the control group at 6 weeks postnatal [35]. The number of women in the intervention group who scored above 13 in the EPDS reduced from 15 to 9 post intervention and the control group reported an increase of women who scored above 13, from 11 to 17 [30]. The studies are summarised in Table 2.

**Table 2:** Description of Included Studies

Country, Author, Date	Study design	Intervention	Depression Assessment Instrument	Personnel	Duration	Format	Location	Evaluation Objective	Data collection method
Pakistan (Rahman, 2007)	Cluster RCT	Cognitive Behavioural Therapy	Schedules of Clinical Assessment in Neuropsychiatry	Lady health workers	16 sessions	Individual	Home/ Community	To develop and evaluate some processes of intervention delivery	4 Focus group discussions 30 In-depth interviews
India (Rath et al., 2010	Cluster RCT	Participatory learning and action cycle (psychoeducation)	Kessler 10	Female Community health workers	20 monthly group meetings (under 2 h)	Group	Home/ Community	Process evaluation of the intervention	244 Focus groups Document reviews Analysis of evaluation forms
China (Gao et al. al, 2012)	Individual RCT	Interpersonal Psychotherapy	Edinburg Post-natal Depression Scale	Midwives	3 sessions (2, 90 min sessions and 1 follow up phone call	Group	Hospital	Post intervention process and outcome evaluations	83 Program satisfaction questionnaires 20 outcome evaluations

### Recipients and provider characteristics

IPT recipients were middle class first time mothers under the age of 35 with uncomplicated pregnancies, PPD symptoms and no family history of psychiatric illness [30]. THP recipients were purposefully selected depressed mothers of low socio-economic status aged 17–40 in their perinatal period [36].The Ekjut study participants were a purposefully sampled group of pregnant women and mothers aged 15–49 who had received the intervention and all group facilitators who provided the intervention [29].

### Recruitment, training and supervision of personnel

The IPT study indicated that personnel received intensive training and supervision on the intervention without giving further details regarding the nature of training and supervision [30]. In the Ekjut study, the personnel received five days of training on participatory communication, how to discuss basic health problems and two days of additional training after six months [29] (no additional information is given on the supervision of the personnel). The THP used Lady Health Workers (LHW) of varying ages and experience who had completed secondary schooling to provide counselling after a two-day workshop and one day refresher training three months after the first training. [28]. Supervision comprised half a day a month in group format and included the discussion of challenges, and brainstorming solutions. Personnel were supervised by a mental health professional and a public health expert [28].

### Content of the intervention

The content of the interventions were CBT for the THP [28], IPT for the IPT study [30], psychoeducation, and problem solving therapy (PST) through participatory stories and problem solving games for the Ekjut study. [29] The Ekjut study emphasised collective problem solving and planning and the intervention was divided into four phases: identify and prioritise problems, plan strategies, implement strategies and assess impact. The intervention was conducted over 20 meetings [29]. Group participants organised meetings in the community where they shared lessons learnt with community members to obtain support for implementing strategies to address their problems in pregnancy and childbirth [29]. The THP used CBT techniques of active listening, collaboration with family, guided discovery and homework applied in LHWs’ routine work of maternal and child health education [28]. The IPT study intervention used lectures and videos as the main methods of delivery [30]. IPT content included psycho-education on the transition to motherhood, obstacles to communication, communication skills, information about PPD, developing social support, identifying potential interpersonal conflict after delivery, and skills for resolving interpersonal conflict.

### The duration of the intervention

The duration of the intervention varied for all three studies; for example, the Ekjut study continued for 3 years and conducted 20 monthly group meetings each lasting under two hours [29]. There is no indication of whether the sessions were antenatal or postnatal and if any women dropped out of the intervention. The THP and IPT studies conducted the interventions both in the antenatal and postnatal phases. The IPT study conducted two group antenatal classes of two hours, and a follow up phone call two weeks after delivery [30]. The THP study conducted a total of 16 sessions during the perinatal period - four weekly individual sessions in the last month of pregnancy, three sessions in the first postnatal month and nine sessions thereafter [28]. None of the studies indicated if and how implementation fidelity was monitored [37].

## Thematic analysis of study findings

### Context

Contextual factors included physical location and accessibility as well as upholding cultural norms.

The THP and Ekjut studies were conducted in rural areas within the community [28, 29] while the IPT study was conducted at a regional teaching hospital without further information being provided on the context [30]. According to the Ekjut team, several challenges arose from the rural context of the trial including: physical isolation of villages, difficulties building rapport with marginalised individuals and dealing with dominant group members and cancellations of meetings during festivals.

As part of the context, the theme of upholding cultural norms for increasing the acceptability of the interventions was apparent in all three studies. The THP study explored socio-cultural aspects of depression from participants’ point of view and aspects of delivering the intervention from LHWs’ point of view [28]. The THP study also referred to the importance of respecting participants’ wishes to observe “chilla” (indoor confinement for 40 days post-delivery) thus not allowing women to do outdoor activities at this stage [28]. The IPT study helped participants understand more about the Chinese post-partum practice of “doing the month” which is a 30 day post-partum period designed to strengthen the mother’s self-esteem. This includes a set of practises such as rest and seclusion, avoiding bathing or washing hair and not touching cold water [30, 38]. The Ekjut study made use of culturally appropriate materials during the meetings [29].

The THP study used the qualitative feedback from interviews with participants, lay workers and primary healthcare staff to further develop their intervention [28]. A number of changes were incorporated into the THP development, such as setting out the steps more clearly, integrating the intervention into the daily work of LHW, encouraging the family to participate in the intervention, calling LHW “trainers” instead of “therapists” and replacing the word “depression” with “mental distress” to avoid stigmatising the women [28]. There was no indication of the contextual challenges that were encountered by the IPT study.

### Implementation

Implementation factors included acceptability of the intervention and characteristics of the personnel delivering the intervention.

### Acceptability of the intervention

Several factors aided the acceptability of the intervention delivered in all three studies. These include characteristics of the personnel (see below for further details), training and supervision of the personnel (discussed above), and the content and duration of the intervention. All these can be heavily influenced by the context of the intervention, such as the cultural practises of ‘chilla’ and ‘doing the month’ described above.

### Characteristics of personnel

The Ekjut and the THP studies used lay health workers [28, 29] and the IPT study used midwives [30]. The THP and Ekjut studies emphasised the recruitment of respected women in the community [28, 29] and the Ekjut study also consulted local leaders for input on selection criteria during the formative part of the study [29]. No additional information is given on the recruitment and selection of the midwives for the IPT study.

## Mechanisms

### Counsellor factors

#### Motivation to conduct the sessions

Most of the THP lady health workers felt that the programme gave their work structure, made them more effective and that it was not a burden to their work. This motivated them to deliver the intervention [28]. The Ekjut facilitators reported feeling motivated and felt that the structured content of the intervention contributed to confidence building.

#### Facilitating trust

The Ekjut study participants indicated that being from the same community and flexibility in content and scheduling facilitated communication and trust within the counsellors. Facilitators felt that trust had been developed when participants started practising what they had learnt from the groups [29]. The THP study noted that trust was built through participants working together with the LHWs [28]. The IPT did not look at the issue of trust.

#### Intervention factors

All three studies reported positive feedback about the intervention from both the recipients and the personnel delivering the intervention.

#### Use of stories and visual aids

All the interventions described both collaboration between the participant and counsellor and the use of visual aids as important aspects of the intervention. These visual aids help to include illiterate individuals by making them active participants of the intervention. The Ekjut study engaged in educational problem solving, storytelling and use of picture cards, games and role plays [29]. Similarly, the THP study used materials such as a health calendar and health corner activities to monitor and encourage healthy behaviour among recipients [28]. The IPT study made use of a lecture and video presentations [30].

#### Understandability of content

Most LHW felt that the intervention was useful and they were able to understand the concepts and explain them to participants [28]. The THP study did not have further information on the specific aspects of the intervention that were helpful to the participants. The IPT study participants were motivated to attend the programme and indicated that it helped them to understand and change their attitude on the Chinese practise of “doing the month” [30].

#### Participant factors

THP study participants rated the intervention as either useful or somewhat useful to them (48% and 47% respectively) [28]. IPT study participants revealed that they learnt more about postpartum depression, the transition into motherhood and communication skills which helped them to form better interpersonal relationships [30]. Ekjut study participants felt that a shared experience through using stories helped them to problem solve and learn more from each other [29]. From the IPT study participants, 61.4% indicated that the programme met their learning needs and 47% indicated that the programme met their expectations [30]. Most IPT participants also indicated that the programme helped them to establish or improve their relationships and all participants generally indicated that the programme enhanced their perceived social support.

## Discussion

This review highlighted evidence from the qualitative process evaluations using the MRC framework to examine the context, implementation and mechanisms of the interventions from three studies. The few articles included in this review highlight the paucity of evidence on qualitative data from process evaluations on task sharing interventions for perinatal depression in LAMICs.

The *context* of implementation highlighted cultural aspects of the participants for all three studies in terms of access to the intervention and intervention delivery [20]. The rural communities in Pakistan [28] (THP) and India [29] (Ekjut) used communal methods of intervention delivery such as inclusion of family members and other community members. The same two studies also made use of stories or illustrations to include illiterate participants and to reduce the stigma associated with depression. Both the THP and Ekjut studies emphasise the importance of observing important cultural practices in order to provide culturally sensitive interventions which concurs with Chowdhary and colleagues who suggest that cultural sensitivity improves the acceptability of interventions [13]. It is important to note that the IPT study only collected their evaluation data from interviews with clients whereas the THP and Ekjut studies also included interviews with people who did not participate in the intervention [28, 29].

When looking at *implementation* the three studies highlight common evidence based task sharing interventions in mental health which are CBT, IPT and psycho-education. The training and supervision of the interventions varied, depending on the contextual factors. There is little information provided about the supervision of facilitators for the Ekjut and IPT studies but the THP study gives details of intensive supervision process which included discussion of problems and brainstorming solutions. The duration of the interventions varied across the studies with the IPT study providing only three sessions while the THP and Ekjut studies delivered 16 and 20 sessions respectively. None of the three studies indicate how implementation fidelity was monitored, examining the fidelity to the intervention can help researcher to see if the intervention is implemented in the way it was intended [37].

Regarding the *mechanisms* of the intervention all three studies reported positive feedback about the intervention from the recipients of the intervention. Several factors appeared to contribute to the perceived effectiveness of the interventions. Intervention related factors such as the content and understandability, counsellor factors such as facilitating trust and motivation to conduct the intervention and participant factors such as motivation to attend the sessions and willingness to learn and change their behaviour, in terms of how they look after their children and relate to other people.

The factor of trust was emphasised in the Ekjut and THP studies. Trust was fostered through aspects such as combining participants and lay health workers from the same community and using cultural inclusion. Most of the THP health workers felt that the programme gave their work structure, made them more effective and that it was not a burden to their work [28]. The Ekjut facilitators reported feeling motivated to help change behaviour of participants and also felt that trust had been developed when participants started practising what they had learnt from the groups [29]. These findings show that motivation to deliver or attend an intervention can be seen as provider and participant mechanisms. We can see that participants view intervention positively when personnel delivering the intervention speak the same language, and that the intervention is educational and uses some form of imagery consistent with local cultural meanings [13]. Process evaluations are helpful because they can help to increase the acceptability of an intervention. For example, the THP study made several changes based on the feedback from their qualitative interviews from their formative work such as ensuring that the terminology was appropriate.

Overall, the systematic review highlights qualitative evidence on task shared interventions which can be linked to the MRC framework categories of context, implementation and mechanisms. Understanding how the three factors relate to intervention delivery is the key to developing future interventions which are culturally appropriate and feasible in LAMICS. The context of the interventions determines the type of personnel and activities that are deemed appropriate as seen in all three studies. Counsellor factors such as motivation to deliver the intervention and facilitating trust help to encourage intervention recipients and intervention factors such as the use of visual aids and understandability of the content facilitate learning in participants and help meet their learning needs and expectations. All these factors make interventions culturally appropriate [13].

### Recommendations

For policy makers, we recommend the use of task sharing psychosocial interventions that are culturally adapted through paying attention to the needs of providers and recipients alike. It is also important to pay attention to the duration of training and mechanisms such as trust which is built over time. Therefore it is important to invest sufficient time in training, supervision and delivery of interventions.

For researchers, it is important to publish more comprehensive qualitative process evaluations following the MRC guidelines in order to aid the development of future interventions. There is limited information specifically focusing on training, supervision and monitoring of fidelity of interventions from the selected studies. This information would be helpful for the replication of the study in other LAMICS. Gaining an in-depth understanding of participant and provider perspectives is useful for the development and evaluation of interventions and applying the MRC framework in process evaluations could yield more effective results.

For lay counsellors we recommend that they be open to discussing the challenges or facilitators that they experienced when delivering interventions as this information is crucial for implementation research. For depressed women in the communities we recommend that additional support and training as peer educators be conducted in line with the recent peer-delivered THP study in Pakistan and India [39].

### Limitations

It is important to be aware of the possibility of publication bias for all identified studies, since we did not include unpublished studies and studies which were not in English. This aspect could limit the potential number of studies included in the review. It would have been helpful to know which aspects of the intervention LHW and participants found to be useful to help us understand the mechanisms involved in the effectiveness of the interventions. We contacted authors of the THP and IPT studies requesting more information on fidelity to the intervention, and training and supervision of personnel. The authors responded however the information that they provided did not shed any new light on these areas as this information was not included in their analyses. We also checked the reference lists of included studies for additional sources of information however no additional information was obtained.

## Conclusion

This review highlights qualitative evidence of process evaluations for task shared interventions for perinatal depression in LAMICS from three studies. There are common mechanisms which can be recommended for successful implementation of interventions, including counsellor factors, intervention factors, and participant factors. More qualitative and comprehensive process evaluations of task shared interventions for perinatal depression are necessary to help us to understand what works and what does not work when implementing a task shared intervention both at the level of the client-provider interaction and the services and systems level. A more comprehensive application of the MRC framework for process evaluations of complex interventions would provide further information, such as fidelity to the implementation of the intervention.

## Additional files

Additional file 1: Search strategy. A document which shows the search terms and search strategy that was used for the study. (DOCX 20 kb)

Additional file 2: Preferred Reporting Standards for Systematic Reviews and Meta-analysis (PRISMA) Statement. A statement detailing the preferred reporting standards for systematic reviews. (DOCX 16 kb)

Additional file 3: Data extraction Table. A data extraction table detailing the information that was extracted from selected studies. (DOCX 15 kb)

Additional file 4: Critical Appraisal Skills Programme (CASP) qualitative data checklist. A checklist for the assessment of qualitative data. (DOCX 12 kb)

## Abbreviations

CBT: Cognitive Behavioural Therapy
HIC: High Income Country
IPT: Interpersonal Therapy
LAMIC: Low and Middle Income Country
MRC: Medical Research council
PPD: Postpartum Depression
PST: Problem Solving Therapy
THP: Thinking healthy programme
