# Supplementary material for: Process evaluations of task sharing interventions for perinatal depression in low and middle income countries (LMIC): a systematic review and qualitative meta-synthesis
Source: BMC Health Serv Res. 2018 Mar 23;18:205. doi: 10.1186/s12913-018-3030-0 (PMC5865346; doi:10.1186/s12913-018-3030-0)
Supplement: Supplementary file 1 — Search strategy. A document which shows the search terms and search strategy that was used for the study. (DOCX 20 kb) [file 12913_2018_3030_MOESM1_ESM.docx]

**Additional File 1: Search Strategy**

**Pubmed**

**PUBMED MesH terms**

**1. Aides, community**

- **Community Health Worker**
- **Health Worker, Community**
- **Health Workers, Community**
- **Worker, Community Health**
- **Workers, Community Health**
- **Community Health Aides**
- **Aide, Community Health**
- **Aides, Community Health**
- **Community Health Aide**
- **Health Aide, Community**
- **Health Aides, Community**
- **Family Planning Personnel**
- **Personnel, Family Planning**
- **Planning Personnel, Family**
- **Village Health Workers**
- **Health Worker, Village**
- **Health Workers, Village**
- **Worker, Village Health**
- **Workers, Village Health**
- **Village Health Worker**
- **Barefoot Doctors**
- **Barefoot Doctor**
- **Doctor, Barefoot**
- **Doctors, Barefoot**
- **Family Planning Personnel Characteristics**

**Perinatal depression**

- **Postpartum Depression**
- **Postnatal Depression**
- **Depression, Postnatal**
- **Post-Natal Depression**
- **Depression, Post-Natal**
- **Post Natal Depression**
- **Post-Partum Depression**
- **Depression, Post-Partum**
- **Post Partum Depression**

**Low and middle Income countries**

- **Countries, Developing**
- **Country, Developing**
- **Developing Country**
- **Least Developed Countries**
- **Countries, Least Developed**
- **Country, Least Developed**
- **Developed Countries, Least**
- **Developed Country, Least**
- **Least Developed Country**
- **Less-Developed Countries**
- **Countries, Less-Developed**
- **Country, Less-Developed**
- **Less Developed Countries**
- **Less-Developed Country**
- **Under-Developed Nations**
- **Nation, Under-Developed**
- **Nations, Under-Developed**
- **Under Developed Nations**
- **Under-Developed Nation**
- **Third-World Countries**
- **Countries, Third-World**
- **Country, Third-World**
- **Third World Countries**
- **Third-World Country**
- **Third-World Nations**
- **Nation, Third-World**
- **Nations, Third-World**
- **Third World Nations**
- **Third-World Nation**
- **Under-Developed Countries**
- **Countries, Under-Developed**
- **Country, Under-Developed**
- **Under Developed Countries**
- **Under-Developed Country**
- **Developing Nations**
- **Developing Nation**
- **Nations, Developing**
- **Less-Developed Nations**
- **Less Developed Nations**
- **Less-Developed Nation**
- **Nation, Less-Developed**
- **Nations, Less-Developed**

**Process Evaluation**

**Task sharing**

1. **Task shifting**
2. **Community health aides/**

**Depression**

1. **Depressive disorders**
2. **Depressive mood**
3. **Distress**

**("community health workers"[MeSH Terms] OR ("community"[All Fields] AND "health"[All Fields] AND "workers"[All Fields]) OR "community health workers"[All Fields] OR ("aides"[All Fields] AND "community"[All Fields] AND "health"[All Fields]) OR "aides, community health"[All Fields]) AND (((("community health workers"[MeSH Terms] OR ("community"[All Fields] AND "health"[All Fields] AND "workers"[All Fields]) OR "community health workers"[All Fields] OR ("aides"[All Fields] AND "community"[All Fields] AND "health"[All Fields]) OR "aides, community health"[All Fields]) AND ("counselling"[All Fields] OR "counseling"[MeSH Terms] OR "counseling"[All Fields])) AND ("depression, postpartum"[MeSH Terms] OR ("depression"[All Fields] AND "postpartum"[All Fields]) OR "postpartum depression"[All Fields] OR ("depression"[All Fields] AND "post"[All Fields] AND "natal"[All Fields]))) OR ((((antenatal[All Fields] AND ("depressive disorder"[MeSH Terms] OR ("depressive"[All Fields] AND "disorder"[All Fields]) OR "depressive disorder"[All Fields] OR "depression"[All Fields] OR "depression"[MeSH Terms])) OR (("prenatal care"[MeSH Terms] OR ("prenatal"[All Fields] AND "care"[All Fields]) OR "prenatal care"[All Fields] OR "prenatal"[All Fields]) AND ("depressive disorder"[MeSH Terms] OR ("depressive"[All Fields] AND "disorder"[All Fields]) OR "depressive disorder"[All Fields] OR "depression"[All Fields] OR "depression"[MeSH Terms]))) OR (perinatal[All Fields] AND ("depressive disorder"[MeSH Terms] OR ("depressive"[All Fields] AND "disorder"[All Fields]) OR "depressive disorder"[All Fields] OR "depression"[All Fields] OR "depression"[MeSH Terms]))) OR (("mothers"[MeSH Terms] OR "mothers"[All Fields] OR "maternal"[All Fields]) AND ("depressive disorder"[MeSH Terms] OR ("depressive"[All Fields] AND "disorder"[All Fields]) OR "depressive disorder"[All Fields] OR "depression"[All Fields] OR "depression"[MeSH Terms]))) AND ("developing countries"[MeSH Terms] OR ("developing"[All Fields] AND "countries"[All Fields]) OR "developing countries"[All Fields]))**

**PsycINFO**

[**process evaluation AND task sharing OR task shifting AND perinatal depression OR antenatal depression OR postnatal depression AND LAMICS**](http://web.b.ebscohost.com/ehost/breadbox/search?term=process%20evaluation%20AND%20task%20sharing%20AND%20perinatal%20OR%20antenatal%20OR%20postnatal%20depression%20AND%20LAMICS&sid=58bff8dd-c48b-4202-bca0-dffb4399bf1c%40sessionmgr115&vid=25)

**((MM "Depression (Emotion)") AND (DE "Community Involvement" OR DE "Community Mental Health" OR DE "Community Mental Health Services" OR DE "Community Counseling" OR DE "Community Services")) OR (DE "Developing Countries")**

**CINAHL**

[**Process evaluation AND task sharing OR task shifting AND perinatal depression OR antenatal depression OR postnatal depression AND LAMICS**](http://web.b.ebscohost.com/ehost/breadbox/search?term=process%20evaluation%20AND%20task%20sharing%20AND%20perinatal%20OR%20antenatal%20OR%20postnatal%20depression%20AND%20LAMICS&sid=58bff8dd-c48b-4202-bca0-dffb4399bf1c%40sessionmgr115&vid=25)

**Maternal depression OR perinatal depression OR postnatal depression OR antenatal depression AND community health worker***

**Scopus**

**Process evaluation AND intervention AND perinatal depression OR postnatal depression Or antenatal depression**

**(TITLE-ABS-KEY(community health workers AND maternal depression AND developing countr*)) AND ((community health AND maternal depression AND developing countr*)) AND (community health worker* AND maternal depression AND developing countr*)**

**Web of Science**

**(Process evaluation AND task sharing OR task shifting AND perinatal depression OR antenatal depression OR postnatal depression AND developing countr*)**

**Refined by: RESEARCH AREAS: ( PSYCHIATRY OR OBSTETRICS GYNECOLOGY OR PSYCHOLOGY OR HEALTH CARE SCIENCES SERVICES OR NURSING OR WOMEN S STUDIES OR ETHNIC STUDIES OR BEHAVIORAL SCIENCES OR SOCIAL ISSUES ) AND LANGUAGES: ( ENGLISH ) AND [excluding] COUNTRIES/TERRITORIES: ( USA OR TAIWAN OR ENGLAND OR BELGIUM OR MALTA OR AUSTRALIA OR CANADA OR SCOTLAND OR AUSTRIA OR SWEDEN OR UK OR NEW ZEALAND OR UNITED ARAB EMIRATES OR NETHERLANDS OR GREECE OR POLAND OR SOUTH KOREA OR ITALY OR NORTH IRELAND OR JAPAN OR GERMANY OR FRANCE OR SINGAPORE OR DENMARK OR ISRAEL OR MALAYSIA OR WALES OR SLOVENIA OR SWITZERLAND OR HUNGARY OR FINLAND OR U ARAB EMIRATES OR QATAR OR PORTUGAL OR KUWAIT OR SPAIN OR ICELAND OR NORWAY OR ESTONIA OR IRELAND )**

**Timespan: All years.**

**Search language=Auto**

**Cochrane library**

**# 1 Attitude of health personnel**

**#2 (MESH) descriptors explode Community health workers**

**#3 Maternal depression**

**#4 Perinatal depression**

**#5 Antenatal depression**

**#6 (MESH) descriptors explode depression, postpartum**

**# 7 #1 AND #2 AND #3 OR #4 OR #5 OR #6**
