# Supplementary material for: Process evaluations of task sharing interventions for perinatal depression in low and middle income countries (LMIC): a systematic review and qualitative meta-synthesis
Source: BMC Health Serv Res. 2018 Mar 23;18:205. doi: 10.1186/s12913-018-3030-0 (PMC5865346; doi:10.1186/s12913-018-3030-0)
Supplement: Supplementary file 3 — Data extraction Table. A data extraction table detailing the information that was extracted from selected studies. (DOCX 15 kb) [file 12913_2018_3030_MOESM3_ESM.docx]

**Additional file 3: Data Extraction Table**

| **Study Site** | **Sample size** | **Inclusion criteria** | **Study Design** | **Intervention type** | **Process Evaluation** | **Main Findings** | **Implementation** | **Mechanisms** | **Context** |
| --- | --- | --- | --- | --- | --- | --- | --- | --- | --- |
| **Rawalpindi, Pakistan** | **30 Perinatally depressed women, 24 LHWs, 6 primary care staff** | **Depressed women, LHWs and Primary healthcare staff** | **Multi-method formative study** | **CBT for depression** | **Post intervention interviews with community health workers and participants of the Thinking healthy programme.** | **Exploring socio-cultural aspects of depression from participant's point of view. Exploring aspects of delivering the intervention from LHW point of view, most health workers felt that intervention was useful and they were able to understand the concepts and explain them to participants. Most did not feel that the programme was a burden to their work.** | **Making intervention easy to follow to include illiterate participants same 3 steps to every session. Not calling the intervention “therapy” but “training”, could prevent stigma of labelling. Difficulty accessing women if agenda was to treat depression. Discussing infant outcomes, window for access to intervention- health promotion and shared goals, more culturally appropriate, intervention involving the whole family, making mothers active participants as opposed to passive recipients in order for changed in thinking to change behaviour .E.g. passiveness is I was told I should take it, versus I should take it because it is important. Heavy workload for LHWs, therefore need to integrate into existing schedules. Patchy services in existing LHW programme due to poor selection of workers, unmotivated health workers, and poor governance- which needed changing for intervention to be effective.** | **Problem perception- depression not a problem needing intervention. Stigma, from being labelled as depressed, talk therapy not material assistance/ or gain. Willingness to learn new childcare practices for optimum child development** | **LHW were already vising mothers in homes, additional training required to facilitate the THP. Making the intervention culturally appropriate, e.g. observing cultural practices such as observing chilla (when a woman rests after giving birth). Culturally appropriate illustrations important** |
| **Jharkhan and Orissa, India** | **244 women's groups, population of 114 141, 18 Group facilitators in intervention areas,** | **Pregnant women** | **Mixed methods process evaluation** | **Participatory learning and action cycle** | **Context, content, and implementation of intervention, potential mechanisms for impact and indication of challenges experienced in the field.** | **6 broad interrelated factors influenced the intervention's impact. 1) acceptability, 2) participatory approach to the development of knowledge skills and 'critical consciousness' 3) community involvement beyond the groups 4) a focus on marginalised communities 5) the active recruitment of newly pregnancy women into groups 6) high population coverage. If key characteristics of participatory interventions with community groups are maintained and adapted to fit the local context then they can influence maternal and child outcomes. In order to scale up these interventions, a detailed understanding of the way in which context affects the acceptability and delivery of the intervention, planned but flexible adaptation and replication of key intervention features and strong support for participatory methods from implementing agencies. Lay health workers as facilitators** | **Acceptability affects the implementation of the intervention, participatory approach leads to critical consciousness, active recruitment of newly pregnant women into the groups, high population coverage,** | **Community involvement beyond the groups, willingness to earn and to change child care practices** | **Focus on marginalised communities, rural communities, limited access to health services,** |
| **China** | **20 participants** | **women who received the intervention** | **mixed methods process evaluation** | **Psycho-education , IPT techniques** | **one on one in-depth interviews with service users** | **IPT conducted by midwives** | **Knowledge, information and skills, interaction with midwife and other women** | **Motivation to attend the programme, positive feedback, better interpersonal relationships, willingness to learn** | **China, chines culture, family oriented,** |
