# Supplementary material for: Process evaluations of task sharing interventions for perinatal depression in low and middle income countries (LMIC): a systematic review and qualitative meta-synthesis
Source: BMC Health Serv Res. 2018 Mar 23;18:205. doi: 10.1186/s12913-018-3030-0 (PMC5865346; doi:10.1186/s12913-018-3030-0)
Supplement: Supplementary file 4 — Critical Appraisal Skills Programme (CASP) qualitative data checklist. A checklist for the assessment of qualitative data. (DOCX 12 kb) [file 12913_2018_3030_MOESM4_ESM.docx]

**Additional file 4: Critical Appraisal Skills Programme Qualitative data checklist**

| **Article(s)** | **Clear statement of aims** | **Appropriate methodology** | **Appropriate research design** | **Detailed, justified, recruitment strategy** | **Appropriate data collection methods** | **Researcher-participant relationship considered** | **Ethical issues considered** | **Rigorous data analysis** | **Clear findings** | **Value of research discussed** |
| --- | --- | --- | --- | --- | --- | --- | --- | --- | --- | --- |
| 2007 Rahman, A. | Y | Y | Y | I | I | Y | Y | Y | Y | Y |
| 2010 Rath et al. | Y | Y | Y | Y | Y | Y | Y | Y | Y | Y |
| 2012 Gao, Luo, Chan | Y | Y | Y | I | Y | y | Y | Y | Y | Y |

**(Y=Yes, N=No, I=Insufficient information)**
